# Experiences of Dementia Caregiving among Korean Americans and Korean Family Caregivers in a Cultural Context

**DOI:** 10.1093/geroni/igaf122.3583

**Published:** 2025-12-31

**Authors:** Ji Yeon Lee, Kyung Hee Lee, Eunhye Hong, Jing Wang, Bei Wu

**Affiliations:** Inha University, Incheon, Republic of Korea; Yonsei University, Seoul, Republic of Korea; Yonsei University, Seoul, Republic of Korea; University of New Hampshire, Durham, New Hampshire, United States; NYU Shanghai, Shanghai, China (People’s Republic)

## Abstract

Many studies have focused on dementia care experiences; however, few have examined these experiences through a cross-cultural lens among individuals sharing the same ethnic background but living in different sociocultural environments. This study aimed to explore how family dementia caregiving experiences were shaped by cultural and societal contexts among Korean-American and Korean caregivers. This international collaborative qualitative study was conducted in the US and South Korea. Participants were family dementia caregivers who provided care to community-dwelling older adults. Researchers interviewed two groups of 11 Korean-Americans in New York and three groups of 14 Koreans in Seoul. Semi-structured interviews explored challenges in dementia care, familial and personal changes due to dementia care, and the impact of culture and social resources on dementia care. Four layers of themes emerged: “Ongoing challenges within the current cultural context,” “support and limitations of the social system,” “the dynamics of family relationships,” and “the impact and life changes.” Eleven categories emerged from four themes and some were unique to each group. For example, “differences and inconveniences experienced as Korean-Americans living in the US” was only from Korean-Americans, whereas “feeling disappointed and regretful toward family” emerged exclusively among Korean participants. This study deepens our understanding of both the differences and similarities in the dementia experiences of Korean-American and Korean family caregivers, who share a cultural heritage but live in distinct societal contexts. The findings highlight the need for culturally sensitive, family-centered approaches to dementia care that address the unique challenges faced by caregivers in different settings.

